# Bacterial delivery of the anti-tumor azurin-like protein Laz to glioblastoma cells

**DOI:** 10.1186/s13568-020-00995-8

**Published:** 2020-03-27

**Authors:** Manar Mansour, Shehab Ismail, Khaled Abou-Aisha

**Affiliations:** 1 Department of Microbiology and Immunology, The German University in Cairo (GUC), Main Entrance Fifth Settlement, Cairo, Egypt; 2 The Cancer Research Institute CRUK Beatson Institute, Glasgow, UK

**Keywords:** Hypoxia, *Salmonella* VNP20009, Azurin-like protein, Glioblastoma

## Abstract

*Salmonella typhimurium* VNP-20009 (VNP) is a non-pathogenic attenuated strain, which, as a facultative anaerobe, preferentially accumulates in hypoxic regions of solid tumors. Here, VNP was utilized as a delivery vehicle of the anti-tumor protein Lipidated azurin, Laz, which is produced by the meningitis-causing bacterium *Neisseria meningitides*. In brain cancer cells, Laz has been demonstrated to induce apoptosis through an interaction with the tumor suppressor protein p53. In this study, the *laz* gene, including its signal sequence, was cloned downstream of a hypoxia inducible promoter (HIP-1), before being electroporated into VNP. Successful ectopic expression and export of the Laz protein by VNP under hypoxic conditions were confirmed by Western blot analysis of the cell-free culture medium. Effective expression of Laz by VNP was investigated in two glioblastoma cell lines: LN-229 and U-373, with the latter line carrying a mutated version of p53; as well as in the breast cancer line MCF-7. Cytotoxicity of the VNP-Laz was assessed by determining the fluorescence of the apoptotic marker caspases 3/7. Compared to the purified Laz, VNP-Laz, significantly induced apoptosis in MCF-7, LN-229 and, to a much lower extent in U-373 cells, suggesting a p53-linked mechanism. Our results might represent a new approach of targeted gene delivery and suggest a potential application in brain tumor therapy.

## Key points

- We investigated the use of VNP as a delivery vehicle of the Neisserial polypeptide Laz, known to cross the blood brain barrier during Neisserial infections, for further augmenting the cytotoxicity of the strain.
- We also restricted the expression of the therapeutic protein, Laz, to hypoxic tumor regions by utilizing the hypoxia inducible promoter HIP-1 upstream of *laz* for selective targeting of hypoxic tumor regions.
- As a proof of principle, we tested our Laz delivery system in two glioblastoma cell lines harboring either wild type- or mutated versions of the tumor suppressor protein p53, under hypoxic conditions.
- The levels of the apoptotic caspase-markers 3/7 were determined to assess potential cytotoxicity of the Laz delivery system. Our results show a clear enhancement of the VNP-Laz strain over VNP, or Laz individually, in an apparently p53-related manner.

## Introduction

Physiological properties of solid tumors significantly determine their response to anticancer therapies. Abnormal vasculature is a key characteristic, which leads to an inadequate and non-uniform vascular network of tumors. This functionally abnormal vasculature within solid tumors occurs when the exponential growth of the tumor outpaces the growth of a supportive blood vessel network. The newly formed blood vessels in tumors are highly irregular and poorly organized, leading to the development of regions of low oxygen tension (≤ 0.1% oxygen), hypoxia, and necrosis at the core of the tumor (Padhani et al. 2007). Systemic delivery of chemotherapeutics is not effective due to the inability to diffuse far beyond the tumor vasculature and developing a suboptimal therapeutic concentration of the cancer drug within the hypoxic areas of solid tumor (Agarwal et al. 2013). Targeted delivery of anti-tumor therapeutic proteins is being developed, however still of limited applicability due to inadequate vasculature especially in areas of hypoxia in solid tumors. Moreover, hypoxic tumor cells are often slow- or non-proliferating and therefore, they show resistance to conventional caner chemotherapeutics (Kim et al. 2009). This situation has urged the search for active or cellular drug delivery vectors that can selectively target, replicate and actively move to hypoxic regions of tumors following systemic administration. Bacterial vectors of various genera have been shown to specifically colonize hypoxic areas of solid tumors following systemic or oral administration in vivo (Baban et al. 2010; Forbes, 2013). The most frequently used bacterial vectors for tumor targeting belong to the genera *Clostridium*, *Bifidobacterium*, and *Salmonella* (Forbes 2013). Bifidobacteria and clostridia are strict anaerobes explaining their preferential localization to hypoxic tumor regions following administration. *S. typhimurium*, a close relative to the most widely used bacterial model organisms *E. coli* and, thus, easy to genetically manipulate, has emerged as an interesting candidate for tumor treatment. *S. typhimurium* is a facultative anaerobic bacterium that, similar to clostridia and bifidobacteria, was shown to accumulate in tumors in vivo in several studies. Although the organisms are, in theory, capable of colonizing aerobic environments, *Salmonella* was shown to selectively accumulate in tumor tissue probably as a consequence of chemotaxis triggered by metabolites such as amino acids and nucleotides, which are released in hypoxic/necrotic regions of tumors and support growth at these sites (Coutermarsh-ott et al. 2017). Both chemotaxis and proliferation were found to be essential for bacterial accumulation (Kasinskas and Forbes 2006). It is also suggested that TNF-α is an important and effective factor in the initial phase of bacterial tumor-colonization, but probably is not the only cytokine involved in bacterial entry into solid tumors (Leschner et al. 2009). However, the exact mechanism by which *Salmonella* cells invade and colonize tumors is not entirely understood. Others authors suggest that tumor invasion and colonization does not require bacterial motility or chemotactic responsiveness. For *Salmonella* sp., the intrinsic tumor-targeting properties have been improved by genetic modification introducing auxotrophies for nutrients that are present in high amounts in tumor tissue and also by generating strains with growth defects under aerobic conditions (Yu et al. 2012; Zhao et al. 2005; Ryan et al. 2006; Mi et al. 2019). *S. typhimurium* is a pathogenic organism that may cause severe side effects such as endotoxemia when wild type strains are administered systemically (Liu et al. 2014). For this reason, *S. typhimurium* vaccine strains have been developed with reduced virulence and improved tumor-targeting properties. This was done by disrupting *purI* gene (a homologue of *purM* of *E. coli*) resulting in an auxotrophy for purines. This made the strain deficient in synthesizing purines, creating a need for external sources of purines for survival and multiplication (Low et al. 1999; Pawelek et al. 1997). Compared to normal cells, tumor cells are richer in purines with adenosine triphosphate concentrations in the level of hundreds of micromolar, whereas in normal cells of healthy tissue concentrations are below detectable levels (Buffon et al. 2007; Pellegatti et al. 2008; Virgilio and Francesco 2012). Moreover, the *msbB* gene was deleted, removing a protein essential for the myristoylation of the Lipid A moiety of the lipopolysaccharide (Broadway et al. 2014). The resulting strain *S. typhimurium* VNP20009, besides being genetically attenuated, displays increased accumulation in tumors by three orders of magnitude, significantly reduced capacity to induce septic shock, and a 10,000-fold increased LD_50_ (Low et al. 1999; Fu et al. 2008).

In this study, *S. typhimurium* VNP20009 was employed to express and secrete the bacterial antitumor protein, azurin-like protein, Laz, a lipid-modified azurin. In pathogenic bacteria, azurin exists in two forms, a periplasmic form in *Pseudomonas aeruginosa* and common in other bacteria, and in a surface-exposed modified form, in gonococci and meningococci (Baarda et al. 2018). In *Neisseria*, azurin is surface displayed due to existence of an extension of a 39-amino acid lipidated peptide, an H.8 epitope in the N-terminal part (Hong et al. 2006). The structural flexibility of the H.8 motif in Laz explains the extracellular location of Laz in *Neisseria*. Being surface exposed enables Laz to bind the key components of brain tumor cells to disrupt their tight junctions and allow entry of Laz inside the tumors to exert cytotoxicity (Hashimoto et al. 2015). Both azurin and Laz possess anticancer activities. Pseudomonal azurin (Paz) showed toxicity against a range of cancers, however, not brain tumors. It was shown that clinical isolates of *P. aeruginosa* secrete Paz extracellularly in response to the presence of various human cancer cells (e.g., melanoma and breast cancer cells) (Mahfouz et al. 2007). In contrast, Laz is produced by *N. meningitidis*, which has the ability to cross the blood–brain barrier to infect brain meninges. It is believed that H.8 component of surface-exposed Laz is essential in facilitating the pathogenic meningococci to cross an entry barrier to attack brain tumors (Hong et al. 2006). As mentioned azurin and Laz exhibit significant cytotoxicity against cancer cells, while little cytotoxicity is observed against normal cells (Punj et al. 2004). They can preferentially enter cancer cells and bind to the tumor suppressor protein p53 (Yamada et al. 2002a, b). This complex formation stabilizes p53 by protecting it from ubiquitination and degradation (Howley et al. 1996), a property that enables Laz to induce apoptosis in cancer cells. Laz also inhibits cancer cell growth through inhibition of the phosphorylation of a receptor tyrosine kinase EphB2 that is often hyper-expressed in cancer cells; in addition to its ability to inhibit angiogenesis (Chaudhari et al. 2007).

In order to restrict expression of Laz specifically to brain tumor cells and not to normal cells, the *laz* gene was cloned under regulation of a hypoxia-inducible promotor system (HIP-1) that was developed by Mengesha (Gene et al. 2006). (HIP-1) was modified from a portion of the endogenous *Salmonella pep*T promoter and was shown to drive gene expression under hypoxia, but not under normoxia. Modifications to the TATA- and FNR-boxes within HIP-1 allowed fine-tuning of gene induction. This way, we took advantage of the hypoxic nature of solid tumors to target the expression of our therapeutic Laz protein specifically to the core of the tumor, which also is the most resistant to chemotherapy. FNR, the Fumarate and Nitrate Reduction regulator, is a transcription factor that senses oxygen availability and activates gene expression under anaerobic conditions following binding to a consensus FNR recognition sequence (Green et al. 1996). Hypoxia leads to iron–sulphur cluster formation, FNR dimerization and enhanced binding to specific DNA sequences (TTGATnnnnATCAA) in target promoters. On binding, FNR recruits RNA polymerase allowing transcription of the downstream gene. When oxygen levels increase the iron–sulphur clusters are disassembled and FNR is released from the target DNA and gene expression ceases (Green 1996; Khoroshilova et al. 2006; Jordan et al. 1997; Kiley and Beinert 2003).

In this study, the FNR binding site (TTGATnnnnATCAA) in the HIP-1 promoter is centered upstream of the transcription start so that the responsiveness to hypoxia is significantly increased, to activate expression of Laz in the attenuated strain of *S. typhimurium*, VNP20009 and investigate its export, cytotoxic, and apoptotic effects on two glioblastoma cell lines, in comparison with its effect on a breast cancer cell line, all grown under hypoxic conditions.

## Material and methods

### Bacterial strains, plasmids and growth conditions

Characteristics of the bacterial strains and plasmids used are listed in Table 1. *Escherichia coli* XL1 was used as the host strain for all recombinant DNA manipulations. *E. coli* BL21 (DE3) was used for hyperexpression and purification of the Laz protein. For manipulation of bacterial strains, *E. coli* cells were grown at 37 C° in liquid Luria–Bertani (LB) medium containing (yeast extract 5 g l^−1^; tryptone 10 g l^−1^; sodium chloride 10 g l^−1^) and on LB agar plates using standard procedures. When needed, the medium was supplemented with ampicillin to a final concentration of (100 mg ml^−1^). Attenuated VNP cells were grown in LB_0_, a modified LB medium that lacks NaCl (yeast extract 5 g l^−1^; tryptone 10 g l^−1^ without NaCl) at 37 °C supplemented with ampicillin (100 mg ml^−1^), when appropriate, using standard procedures (Low et al. 1999).

**Table 1:** Bacterial strains

Bacterial strain	Genotype or phenotype	Reference/source
*S. typhimurium*		
*S*. *typhimurium* VNP 20009	(*msbB*^−^) auxotrophic (*purI*^−^)	A kind gift from Prof. Brooks Low
VNP-Laz	*S*. Typhimurium VNP20009, pBsKSII-HIP-*laz, amp*^*R*^	This study
*E. coli*		
*XL1*-*Blue*	*recA1*, *endA1 gyrA96* thi-1 *hsdr17 sup E44 relA1* lac^q^ ZΔ M15 Tn 10 (Tet^r^)]	Stratagene
BL21 (DE3)	*fhuA2 [lon] ompT gal (λ DE3) [dcm] ∆hsdS, λ DE3 *=* λ sBamHIo ∆EcoRI*-*B int::(lacI::PlacUV5::T7* *gene1) i21 ∆nin5*	NEB

VNP strains harboring the recombinant plasmids were grown overnight in LB broth at 37 °C in a shaking incubator. In order to determine the bacterial cell count, 100 µl of culture was serially diluted and plated onto LB agar. Bacterial colonies were counted after overnight incubation at 37 °C. Aerobic conditions were obtained by vigorously shaking the culture (200 rpm/min). For anaerobic induction experiments, cultures were incubated in a hypoxic chamber (Oxoid™ AnaeroJar™ 2.5 l—Thermo Fisher Scientific, UK) at 37 °C with shaking.

Competent *E. coli* BL21 (DE3) was used for over-expression of recombinant Laz protein. For protein expression and purification, bacterial cultures were grown in LB broth, under vigorous shaking at 200 rpm, either overnight at 20 °C or grown for 5 h at 37 °C. Recombinant protein expression was induced using 0.3 mM isopropyl b-d-thiogalactoside (IPTG).

### DNA manipulation and cloning

#### Construction of lipidated azurin Laz-expressing carrier under the control of hypoxia inducible promotor

Genomic DNA of *N. meningitidis* strain H44/76 [B: 15:P1.7,16: ST-32; invasive isolate from Norway (1976); is a kind gift from R. Sanjay; University of Massachusetts, USA]. The therapeutic transgene, *laz*, was amplified from *N. meningitidis strain* H44/76 chromosomal DNA using primer pair *LAZ*-*FOR* (5′-GCATT**GAATTC**AAGGAGATTTGTTATGAAAGCGTATC-3′); *the EcoRI* site is emboldened and underlined), and *LAZ*- *REV* (5′-GCTA**GTCGAC**TTAATCGACCAAAGTCACTTTGC-3′; the *Sal*I site is emboldened and underlined); Invitrogen, Heidelberg, Germany). Primers used in this study were designed using the published sequence of *laz* gene [EMBL: STPEPT, AC:]. Restriction endonucleases and DNA-modifying enzymes were purchased from New England Biolabs (NEB Ltd, Ipswich, UK) and used according to the manufacturer recommendations. The PCR product was digested by *EcoR*I HF and *Sal*I (NEB) sequentially. The fragment was then cloned into pBsKSΙΙ vector double digested using the same restriction enzymes and ligated using T4 DNA ligase.

The hypoxia-inducible promoter HIP was synthesized chemically as two complementary single strands HIP-1 and HIP-2 (Invitrogen, Heidelberg, German). Equal molar amounts of the two strands were mixed, cooked at 95 °C for 10 min and allowed to anneal at room temperature. *Sma*I and *EcoR*I HF then double digested the HIP promoter sequentially. The digest was then cloned into the *Sma*I–*EcoR*I site of pBsKSII-*laz*. The construct was designed to have the *laz* gene (NMB 1533) downstream of the HIP promoter in frame. The construct pBsKS-II–HIP-*laz* was then transformed into chemically competent *E. coli* XL1 cells. Positive transformants were confirmed by colony PCR using HIP-FOR (5′-CCCGGGATAAAATTGATCTGAAT-3′) as a forward primer and LAZ- REV (5′-GCTAGTCGACTTAATCGACCAAAGTCACTTTGC-3′) as a reverse primer (Invitrogen).

The construct (pBsKSII-HIP-*laz*) was also verified by Sanger’s sequencing using the same primer pair. The construct was then electroporated into VNP pre-grown to mid-log phase (A_600_ of 0.75), harvested at 4 °C. Cells were made electro-competent after serial washes in ice-cold 1 mM HEPES and 10% glycerol, followed by re-suspension in 10% glycerol at 2.5 × 10^10^ cells ml^−1^. Electroporation was performed in 0.2 cm (cuvettes after mixing 40 μl) competent cells with 25 ng plasmid DNA using Amaxa (Lonza) Micropulser, with settings of field strength of 12.5 kV cm^−1^, 25 µF capacitance, and 200 Ω resistance. Bacteria were selected and maintained in LB_0_ media supplemented with 100 µg µl^−1^ ampicillin. The transformed strain was designated as VNP-Laz.

#### Cloning, expression and purification of recombinant Laz-6xHis

*laz* (NMB 1533) was amplified from *N. meningitidis* strain H44/76 chromosomal DNA using primer pair LAZ-FOR (5′ GG**AATTCC**ATATGTCTCAAGAACCTGCCGCGCC 3′; the *NdeI* site is emboldened and underlined) and LAZ-REV (5′ CCG**CTCGAG**ATCGACCAAAGTCACTTTGCCG 3′; the *XhoI* site is emboldened and underlined). The 496 bp amplicon encoded Laz without the N terminal Cys residue and also lacked the stop codon was cloned into the *Nde*I–*Xho*I sites of pET-21a (Novagen) that introduces a C-terminal 6× His tag. Plasmids with insert, now called pET-21a-*laz*-His, were transformed into *E. coli* XL1, purified and transformed into the expression strain *E. coli* BL-21(DE3). Transformed cells were selected on LB medium supplemented with ampicillin to a final concentration of 100 µg ml^−1^. Laz expression was induced with 0.3 mM IPTG, and the recombinant protein was purified from inclusion bodies after lysis of bacteria with B-PER lysis buffer (Pierce) supplemented with lysozyme (200 mg ml^−1^), followed by nickel-affinity chromatography using ÄKTA Prim Plus. A Ni–NTA (nickel nitrilotriacetic acid) purification 1 ml column (His Trap FF crude; GE Healthcare) was equilibrated with 5 ml of binding buffer, loaded with the supernatant and washed with 15 ml binding buffer. The column was eluted using a step elution protocol at (250 mM) imidazole. The ÄKTA eluted fractions were collected according to the chromatogram given by ÄKTA prim view program at OD_280_ nm. The collected fractions were desalted through a dialysis tubing cellulose membrane (Sigma). The recombinant protein was analyzed for purity by Coomassie blue staining, and the presence of the His-tag was confirmed using Western blot analysis with an anti polyhistidine mAb (Sigma).

### Cell lines and culture media

The human breast cancer MCF-7 cell line (ATCC HTB-22) and the brain tumor U-373 (U-373 MG (Uppsala); ECACC 08061901) and LN-229 (ATCCCRL-2611) human glioblastoma astrocytoma cell lines were maintained as monolayer cultures in RPMI and DMEM culture media, respectively, supplemented with 10% (v/v) heat inactivated fetal bovine serum FBS (LONZA, Germany) and 100 units ml^−1^ penicillin and 100 µg ml^−1^ streptomycin. All cells were grown at 37 °C in 5% CO_2_, and the growth was monitored using light microscopy (Micromaster, Fisher Scientific, UK).

For seeding, cells were harvested from sub-confluent cultures, with 0.25% (w/v) trypsin-0.02% EDTA. Trypsin was neutralized with medium containing 10% fetal bovine serum, washed in Ca^2+^/Mg^2+^-free phosphate buffered saline (PBS) and re-suspended in fresh medium. Viability of cells was determined using 0.5% trypan blue and direct microscopic count using a hemocytometer.

### Production and cytotoxicity of the purified Laz

The Laz protein was purified and quantified from the overproducing *E. coli* strain BL1(DE3) as described above. Responsive induction of apoptosis was determined by Caspase-3/7 assay (Promega, UK) according to the manufacturer’s instructions. Briefly, cells were seeded into 96- well white culture plate at a density of 5 × 10^3^ cells per well in 100 µl serum-free medium and incubated at 37 °C in 5% CO_2_. After overnight incubation, the supernatant was removed and fresh medium containing the purified Laz protein was added to the cells at various concentrations (0.1, 1 and 5 µg ml^−1^). After incubation for 24 h, 100 µl of Apo-ONE Caspase-3/7 reagent were added to each treatment, the plate was covered and incubated at room temperature under continuous shaking at 350 rpm for 30 min. The produced fluorescence by each treatment was measured after excitation wavelength of 485 nm and an emission wavelength of 530 nm using a Perkin Elmer victor^3^V plate reader. Ovalbumin was used as a mock protein control. A blank containing (caspase reagent and cell free culture medium) and a negative control containing (caspase reagent and untreated cells) were also prepared in the same plate.

### Export of Laz under hypoxic conditions by *S. typhimurium* VNP-Laz

To confirm that *S. typhimurium* VNP 20009 transformed with pBsKSII -HIP-*laz,* expresses and exports detectable Laz protein under hypoxic conditions only, cells were grown in LB_0_ broth supplemented with ampicillin at a final concentration of 100 µg ml^−1^ under both normoxic and hypoxic conditions. The concentration of extracellular protein in the medium was estimated by measuring the absorbance at 280 nm and equivalent concentrations were analyzed by sodium dodecyl sulfate polyacrylamide gel electrophoresis (SDS-PAGE). Samples were loaded onto SDS-PAGE, then transferred using standard western blotting procedure and probed with anti-Lip/H.8 mAb2C3 monoclonal antibody (diluted 1:2 in PBS). Membrane-bound IgG was detected using alkaline phosphatase conjugated anti-mouse IgG (secondary antibody; Santa Cruz Biosciences, UK) at 1:1000 dilution in 5% (w/v) skimmed milk powder blotting grade (Roth, Germany). Immune complex was visualized by chemiluminescence using (BCIP/NPT) liquid substrate (Abcam, UK).

### Cytotoxicity of VNP-Laz under hypoxic conditions

Caspase-3/7 assay was performed to determine apoptosis induction by the constructed strain towards different types of cancer cell lines. Induction of apoptosis and consequently activation of caspase as a result of infection of the cancer cells by the constructed strain VNP-Laz was done in comparison with a Laz-free VNP.

To confirm that *S. typhimurium* (VNP-laz) produces and exports a cytotoxic form of Laz under hypoxic conditions, the human breast cancer MCF-7 cells as well as brain tumor U-373 cells and LN-229 were co-cultured with increasing concentrations of VNP-Laz under hypoxic conditions. Cells were seeded at a density of (5 × 10^3^ per well) in 100 µl of serum free medium supplemented with 100 µg ml^−1^ ampicillin in a 96 well opaque white clear bottom culture plate. VNP cells were grown to the mid-logarithmic phase under hypoxic conditions before infecting the cell lines. After overnight incubation, the supernatant was removed and fresh medium containing bacterial cells at various concentrations (10^4^, 10^6^ and 10^8^ colony forming units, CFU) were added to the adherent cells. The bacterial–cell lines co-cultures were incubated at 37 °C, in (Oxoid™ AnaeroJar™ 2.5 l—Thermo Fisher Scientific, UK) for 24 h (Imhof and Heinzer 1996), after which time 100 µl of Apo-ONE Caspase-3/7 reagent was added to each well.

## Results

### HIP-1 promoter drives hypoxia specific Laz expression by *S. typhimurium* VNP20009

To confirm that VNP-Laz expresses and exports detectable Laz under hypoxic conditions, *S. typhimurium* VNP20009 expressing-Laz transformed with pBsKSII-HIP-*laz* was grown for 24 h under hypoxic or normoxic conditions. The cell-free media were then collected, concentrated for 6 h (Eppendorf Concentrator Plus, Germany) and probed onto an immunoblot with monoclonal antibodies anti-Lip/H.8 mAb2C3. Purified Laz was used as a positive control. Pelleted bacterial cells were suspended in water and 5× SDS gel-loading buffer, cooked at 95 °C for 5 min before loading onto the 12% SDS-PAGE gel.

**Fig. 1:**
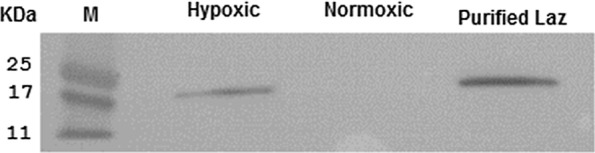
*S. typhimurium* VNP 20009 expressing Laz (VNP-Laz) was cultured under normoxic or hypoxic conditions for 24 h, the media were then collected concentrated and probed onto an immunoblot with anti-Lip/H.8 mAb 2C3

Immunoblotting using anti-Lip/H.8 mAb 2C3 serum showed that Laz was only detected when VNP-Laz were grown under hypoxic conditions and to a much lower extent under normoxic conditions as shown in (Fig. 1).

**Fig. 2:**
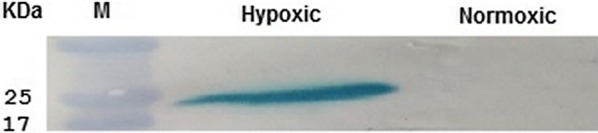
Western blot analysis of VNP-Laz cell pellets probed onto an immunoblot with anti-Lip/H.8 mAb 2C3

We also noticed that the pelleted bacterial cells VNP-Laz (*S. typhimurium* VNP20009 expressing-Laz transformed with pBsKSII-HIP-*laz*) still contain considerable amount of our therapeutic protein Laz bound to the cells. Under normoxic conditions, however, no or negligible amount of Laz was detected in the bacterial cell pellets (Fig. 2).

### Cytotoxicity of Laz

Purified Laz was shown to enter human cancer cells, stabilize p53 to enhance its intracellular level, and induce cancer cell death in vitro (Apiyo and Wittung-Stafshede 2005). Thus, if appropriately delivered, Laz could be used to induce apoptosis in tumor cells.

The experiment was designed so that the developed VNP-Laz strain of *S. typhimurium* infects three different cancer cell lines; namely; breast cancer cell line MCF-7 as a representative of non-brain cancer cells with a wild type p53; brain cancer cell line U-373 as a representative of glioblastoma cells with mutated p53; and LN-229, glioblastoma cells with wild type p53. Such a design aimed at testing the potential of the constructed VNP-Laz strain of attenuated *Salmonella* to deliver a cytotoxic form of Laz to brain tumor cells as well as non-brain tumor cells; confirming the capacity of the surface exposed Laz with its H.8 epitope to disrupt an entry barrier and enabling bacterial cells to enter brain tumor cells and induce apoptosis through interaction with the tumor suppressor protein (p53).

To show that expressed and released Laz by *S. typhimurium* VNP-Laz was cytotoxic and able to induce apoptosis in cancer cell lines, VNP-Laz cells were co-cultured with either breast cancer cells MCF-7, glioblastoma cells LN-229 or U-373 in hypoxic conditions followed by fluorometric assay of caspase 3/7 activity for assessment of apoptosis that is Laz-induced.

**Fig. 3:**
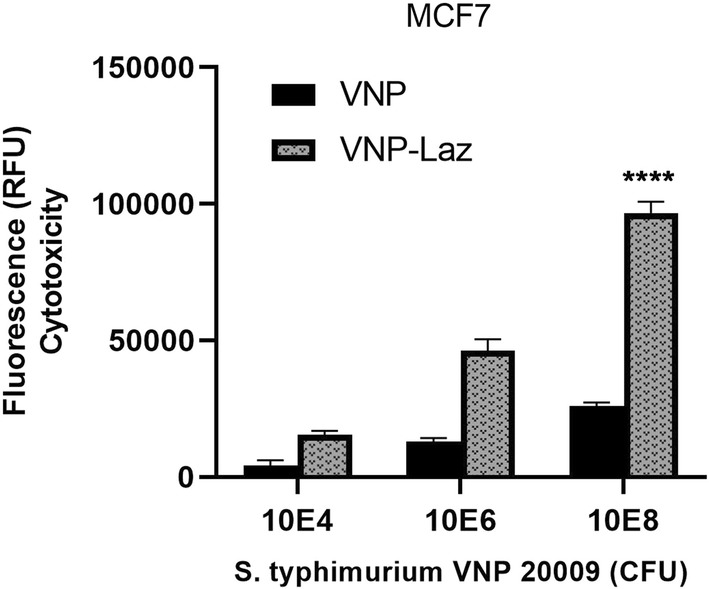
Cytotoxic effect of the *S. typhimurium* VNP20009 expressing Laz (VNP-Laz) on tumor cells in vitro. Monolayers of breast cancer cells MCF-7 were co-cultured with increasing bacterial densities (104, 10^6^, or 10^8^ cfu) of VNP alone or VNP-Laz under hypoxic conditions for 24 h. Apoptosis was assessed using caspase 3, 7 homogenate assay. All data are shown as mean ± s.e.m and represent data from three independent experiments n = 3. *P < 0.0001 with respect to the control groups at the same concentration

**Fig. 4:**
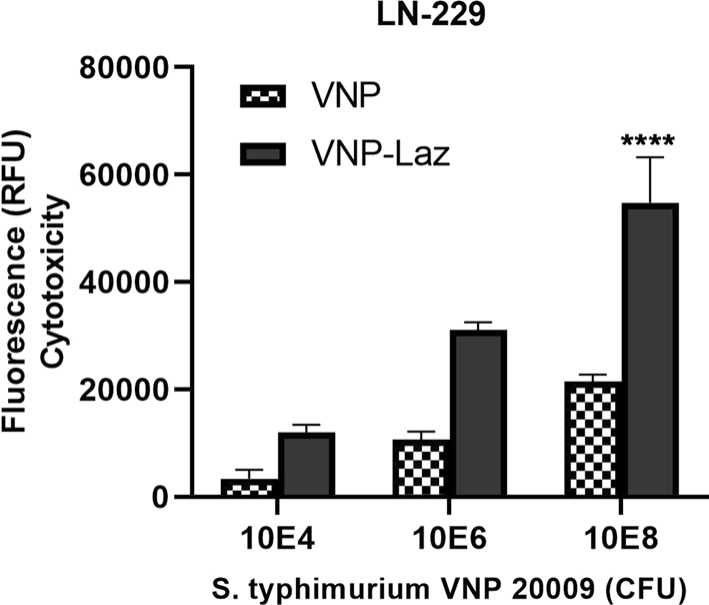
Cytotoxic effect of the *S*. *typhimurium* VNP 20009 expressing Laz (VNP-Laz) on tumor cells in vitro. Monolayers glioblastoma cell line LN-229 (WT p53) were co-cultured with increasing bacterial densities (10^4^, 10^6^, or 10^8^ cfu) of VNP alone or VNP-Laz under hypoxic conditions for 24 h. Apoptosis was assessed the same method of breast cancer cell line for comparison. All data are shown as mean ± s.e.m and represent data from three independent experiments n = 3. *P < 0.0001 with respect to the control groups at the same concentration

**Fig. 5:**
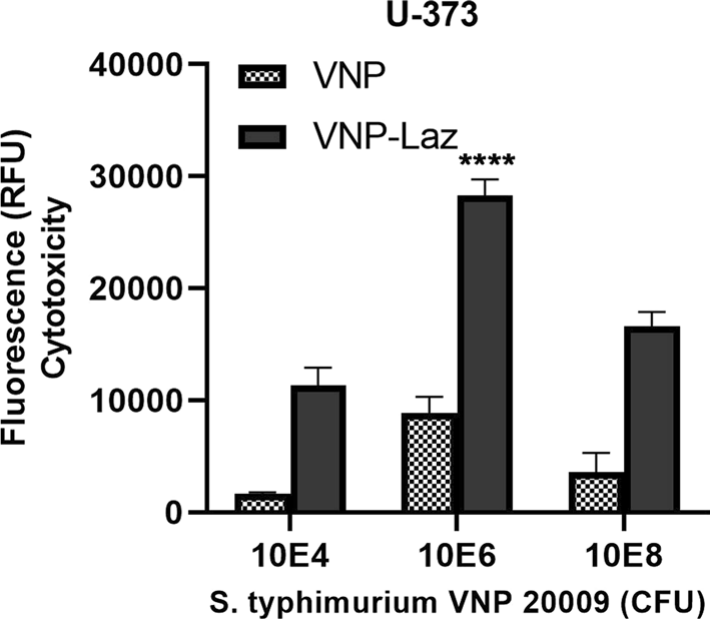
Cytotoxic effect of the *S*. *typhimurium* VNP20009 expressing Laz (VNP-Laz) on tumor cells in vitro. Monolayers glioblastoma cell line U-373 (carrying a mutated p53) were co-cultured with VNP alone, or VNP-Laz using the same bacterial densities mentioned above, under hypoxic conditions for 24 h. Apoptosis was assessed as mentioned above. All data are shown as mean ± s.e.m and represent data from three independent experiments n = 3. *P < 0.0001 with respect to the control groups at the same concentration

As the number of bacteria in the inoculum increased the intensity of fluorescence increased after 24 h of co-incubation with breast cancer cells MCF-7 (Fig. 3); glioblastoma cells LN-229 (Fig. 4) and U-373 (Fig. 5). VNP-Laz caused significantly higher level of apoptosis induction and hence fluorescence compared to that induced by VNP alone. Most notable was the difference in the cytotoxicity of the purified Laz and VNP-Laz between glioblastoma and breast cancer cells.

The cytotoxic effect of VNP-Laz on U-373 was dose-dependent and significantly higher than the VNP control (Fig. 5). The highest level of apoptosis was measured with 10^6^ cfu of either VNP-Laz or VNP alone, suggesting that the adhesion of *Salmonella* cells to the glioblastoma cells probably follow saturation kinetics, with maximum number of bacterial cells per cell, which implies that, there are a specific number of bacterial adhesion sites available on the surface of the host cell.

**Fig. 6:**
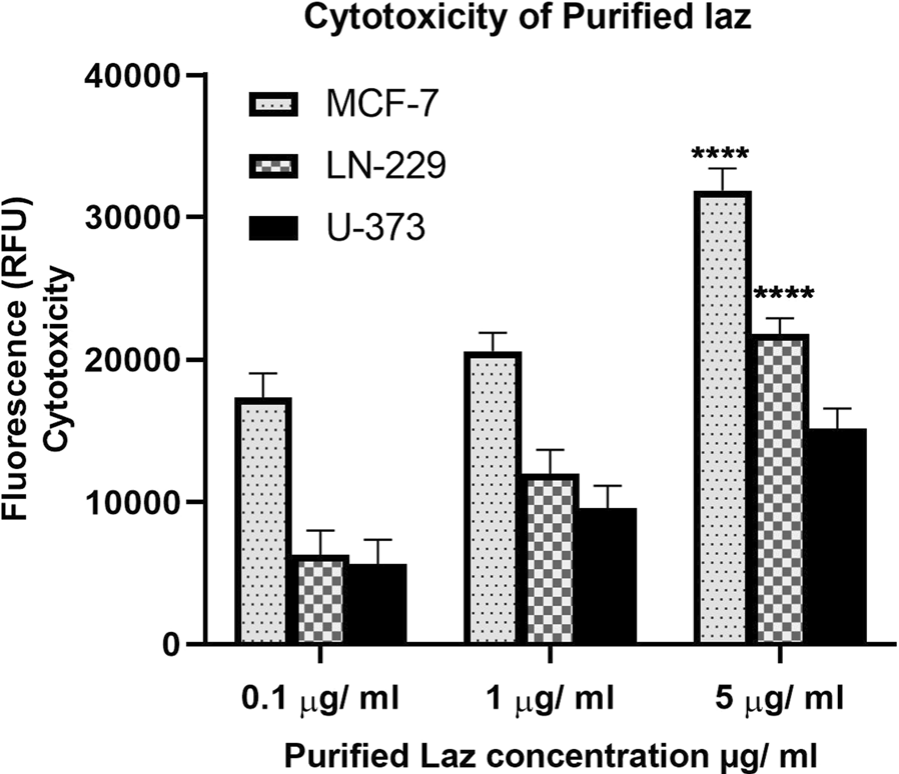
Cytotoxic effect of the purified Laz on tumor cells in vitro. Monolayer of MCF-7 Breast cancer cell line and glioblastoma cell lines U-373 and LN-229 were incubated with increasing concentrations (0.1, 1, 5 μg ml^−1^) of purified Laz protein or a control protein (Ovalbumin) for 24 h. Apoptotic activity was assessed with respect to the control group at the same concentration *P < 0.0001

When the same cancer cell lines (MCF-7, LN-229, U-373) were exposed to purified Laz protein (≥ 1 µg ml^−1^), cancer cells showed a marked increase in caspase 3/7 activity (Fig. 6), indicating induction of apoptosis, albeit lower than that induced by VNP-Laz. This shows the efficiency of the vehicle strain to express and export Laz to levels higher than those attained by the used concentrations of purified Laz. The effect of Laz was dose-dependent in both breast cancer and glioblastoma cells, although lower in glioblastoma U-373 than in both MCF-7 and LN-229 cells, suggesting that induction of apoptosis is p53-mediated. It was also observed that VNP alone (at a cell density of 10^8^ cells) showed a similar p53-dependent cytotoxicity profile to that shown in presence of Laz, albeit with reduced potency (see Figs. 3, 4 and 5).

The enhanced level of apoptosis induction by VNP-Laz clearly shows that a functional Laz was expressed and exported from the bacterium under hypoxic conditions.

## Discussion

The use of bacteria as anticancer agents might have numerous advantages over other therapeutic approaches. Bacterial therapies are taking advantage of microbial metabolism, motility and sensitivity. One of the most important drawbacks, relevant to almost all chemotherapeutic and biological treatments, is limited accessibility of the tumor tissue to passively-distributed therapeutics. In both cases, small molecule drugs, as well as larger molecules such as cytokines, antibodies or viruses, the therapeutic agent diffuses from the blood stream into the periphery, with no transport system that could cross biological barriers, or facilitate preferential accumulation in the tumor tissue. In fact, systemic delivery of passive therapeutics builds relatively large drug concentrations in the bloodstream versus relatively low drug concentrations in the tumor, resulting in limited efficacy and increased extratumoral toxicity (Singh et al. 2008). Bacteria facilitate site-specific treatment that is characterized by being highly concentrated on the tumor and safe to normal tissues. Despite the preclinical success as oncolytic vectors, *Salmonella*, did not show the same efficacy in human studies. Very limited data are available for *Clostridium* in patient however they seemed to be efficient (Theys and Lambin 2015). *Salmonella enterica* serovar Typhimurium (*S. typhimurium*), a facultative anaerobe, has already been utilized as an anti- cancer agent and has been shown to selectively target tumors after intravenous injection (Low et al. 1999). Tumor-targeting *Salmonella* strains that have been developed are both limited in their pathogenesis and have mutant lipopolysaccharide (LPS), which reduces induction of septic shock (due to a deletion in the msbB gene), and yet have high-level intratumoral replication (due to a deletion in the purl gene). *S. typhimurium* VNP20009, attenuated strain had been developed with the properties desirable for a live Gram-negative bacterial anticancer agent with applications in humans (Toso et al. 2002).

Although oncolysis and destruction of most of the tumors was demonstrated, the outer rim of the solid tumor was shown to be clear of bacterial colonization with no oncolytic activity, leading to regrowth of tumors (Wei et al. 2008; Leschner et al. 2009). As a result, this imposes a need to develop an improved tumor—targeting *S. typhimurium* strain that can kill primary and metastatic cancer without toxic effects to the host and without the need for combination with toxic chemotherapy. We find it attractive to take advantage of the natural tendency of *S. typhimurium* to preferentially target tumors over other tissues, and combine it with the use of a promoter that preferentially becomes induced in the tumor environment, which may allow exquisitely tumor—specific expression of a therapeutic fusion protein on the surface of, or secreted by *S. typhimurium* for highly selective and potent tumor therapy (Arrach et al. 2008; Arrach et al. 2010; Leschner et al. 2012; Deyneko et al. 2016).

Although this attenuated strain of *S. typhimurium* has an impressive tumor/liver ratio of 10,000:1, they were present in some normal tissues when injected systemically, which could result in extra-tumoral gene expression (Ryan et al. 2009). It was important to restrict the expression of the therapeutic gene to hypoxic areas within tumors and minimize expression in normal; normoxic tissues. That was achieved by controlling the expression of our therapeutic protein by putting it under the control of the hypoxia inducible promotor (HIP-1) developed by Mengesha (Gene et al. 2006). In which the -10 TATA-box was modified to its consensus sequence in order to maximize the inducibility to achieve optimal expression levels of the therapeutic gene. The specificity was further improved by mutating the FNR binding site to the consensus sequence (TTGATnnnnATCAA). Our results show that HIP-1 successfully induced expression of the therapeutic protein only under hypoxic conditions, however we also observed that much of the protein was still associated with the bacterial outer membrane. Which highlights the need of using a more powerful secretion system for improved export of our therapeutic protein.

In this study, we used *Salmonella* VNP20009 as a delivery vehicle for an anti-cancer bacterial protein, azurin-like protein, Laz. Lipidated azurin, Laz, known to be produced by many gonococci and meningococci (Trees and Spinola 2019; Hayashi and Wu 1990). Since meningococci like *N. meningitidis* are known to cause meningitis by invading the brain meninges, and since azurin is known to have anticancer activity (Punj et al. 2004), the H.8 component of surface-exposed Laz might be involved in facilitating the pathogenic meningococci to cross an entry barrier to attack brain tumors. A property that encouraged us to explore its therapeutic capacities against glioblastoma.

Azurin was shown to enter preferentially to cancer cells (Yamada et al. 2005) (Taylor et al. 2009) and stabilize tumor suppressor p53, increasing the expression of pro-apoptotic Bax and Bax-dependent apoptosis in cancer cells (Punj et al. 2004) (Yamada et al. 2002a, b). In addition to having anti–angiogenic activity, leading to cancer growth inhibition, azurin also inhibits cancer cell growth through interference with the phosphorylation of receptor tyrosine kinase EphB2 that is often hyper-expressed in cancer cells.

Cytotoxicity results provide important evidence that attenuated *S. typhimurium*-VNP0009-mediated Laz delivery can induce significant cytotoxicity and cell apoptosis in glioblastoma cells; fluorescence and hence cytotoxicity levels determined for the VNP-Laz delivery system were higher than those attained using either, VNP or the purified Laz protein, individually.

It is also worth mentioning that the addition of VNP alone (at a cell density of 10^8^ cells) to the same cell lines showed a similar p53-dependent cytotoxicity profile to that observed in presence of Laz, however with reduced potency. This observation agrees with previous reports suggesting that VNP administration activates p53, probably by acetylation involving a virulence factor called AvrA (Wu et al. 2010; El-Bahy et al. 2013). Consequently, the activated p53 induces cell cycle arrest and triggers apoptosis.

In summary, we have developed a successful system of tumor-targeted delivery of a therapeutic apoptosis- inducing protein using the attenuated strain of *S. typhimurium* VNP20009 as a delivery vector. Moreover, we have shown that this system effectively delivers a cytotoxic form of a bacterial anti-tumor Neisserial azurin like protein, Laz, which significantly induced apoptosis in glioblastoma LN-229(WT p53) compared to the U-373 (mutated p53) and breast cancer cells MCF7(WT p53), confirming that the induction of apoptosis is p53-mediated. Thus, this new system represents a promising strategy for delivering gene therapy to largely inaccessible areas of solid tumors. These results might represent a new approach of targeted gene delivery and suggest a potential application in brain tumor therapy.

## Data Availability

Materials described in the manuscript, including all relevant raw data, will be freely available.
